# The effect of Dipeptidyl peptidase 4 (DPP-4) inhibitors on hemoglobin level in diabetic kidney disease: A retrospective cohort study

**DOI:** 10.1097/MD.0000000000034538

**Published:** 2023-08-11

**Authors:** Lingfeng Zeng, Gordon C.K. Chan, Jack K.C. Ng, Winston W.S. Fung, Kai-Ming Chow, Cheuk-Chun Szeto

**Affiliations:** a Carol & Richard Yu Peritoneal Dialysis Research Centre, Department of Medicine & Therapeutics, Prince of Wales Hospital, Shatin, Hong Kong, China; b Li Ka Shing Institute of Health Sciences (LiHS), Faculty of Medicine, The Chinese University of Hong Kong, Shatin, Hong Kong, China.

**Keywords:** anemia, chronic kidney disease, erythropoietin

## Abstract

Anemia typically develops early in the course of diabetic kidney disease (DKD). There are data to show that dipeptidyl-peptidase-4 (DPP-4) inhibitors affect hematopoietic growth factor activity and hemoglobin level. We retrospectively reviewed 443 DKD patients who were started on DDP-4 inhibitor therapy in 2019. Their hemoglobin level at baseline (6–12 months before treatment), pretreatment (0–6 months before treatment), and post-treatment periods (within 6 months after DPP-4 inhibitor), concomitant estimated glomerular filtration rate (eGFR), HbA1c, peripheral blood white cell and platelet counts were reviewed. The severity of kidney failure was classified according to the Kidney Disease: Improving Global Outcomes stages. The hemoglobin level had a small but significant decline from 11.98 ± 2.07 to 11.87 ± 2.12 g/dL from pretreatment to post-treatment period (paired Student *t* test, *P* < .0001). From the pre- to post-treatment period, the decline of hemoglobin level was 0.10 ± 0.89 g/dL, which was significantly less than that from baseline to pretreatment period (0.24 ± 0.90 g/dL, *P* = .0008). The change in hemoglobin level had a positive correlation with the change in HbA1c level (*R* = 0.218, *P* < .0001), but did not correlate with the type of DPP-4 inhibitor or pretreatment eGFR. There was no significant change in peripheral blood white cell or platelet count during the same period. DPP-4 inhibitor ameliorates hemoglobin decline in DKD. The effect of DPP-4 inhibitor on hemoglobin is statistically significant but clinically modest, and did not correlate with the concomitant change in kidney function.

## 1. Introduction

Diabetic kidney diseases (DKD) is one of the most serious complications of diabetes.^[1]^ Approximately 30% to 40% of patients with type 1 diabetes and 20% of patients with type 2 diabetes develop DKD.^[2,3]^ In the past 2 decades, DKD has become the most common cause of end-stage renal disease around the world.^[4,5]^

Although anemia is a common complication of chronic kidney disease (CKD) irrespective of the underlying cause,^[6,7]^ it is generally more severe and occurs at an earlier stage of renal failure in DKD.^[8]^ Anemia is an independent contributor to the pathogenesis and progression of other diabetes-related complications, especially left ventricular hypertrophy.^[9]^ In diabetic patients, correction of anemia improves the quality of life and might delay the progression of other diabetic complications.^[9]^

In the past 2 decades, dipeptidylpeptidase-4 (DPP-4) inhibitors are increasingly used for the treatment of diabetes.^[10]^ DPP-4 inhibitors are orally bioavailable compounds that prevent the cleavage of glucagon-like peptide-1, which is an incretin hormone with potent glucose-dependent insulinotropic actions, trophic effects on the pancreatic β-cells, and inhibitory effects on gastrointestinal secretion and motility, with the end result of lowering plasma glucose level.^[11,12]^ In essence, DPP-4 inhibitors indirectly stimulate glucose- dependent insulin secretion and suppress glucagon secretion from pancreatic α-cells by increasing endogenous glucagon-like peptide-1 levels.^[12]^

More recently, the effect of DPP-4 on the hematopoietic system is increasingly recognized. DPP-4, also known as CD26, is a peptidase expressed on the cell surface of most hematopoietic cells.^[13]^ DPP-4 cleaves a wide variety of substrates, including the chemokine stromal cell-derived factor-1 and, more importantly, the N-terminal of erythropoietin.^[14]^ A previous study showed that hematopoiesis after radiation or chemotherapy was enhanced in DPP-4 knock-out mice or mice receiving an orally active DPP-4 inhibitor.^[14]^ However, to the best of our knowledge, there is no human study on the relation between DPP-4 inhibitor therapy and anemia in DKD patients. We hypothesize that DPP-4 inhibitor therapy prevents the decline in hemoglobin level in DKD patients. In the present study, we examined the change in hemoglobin level in a large cohort of DKD patients who were started on DPP-4 inhibitor treatment.

## 2. Patients and methods

### 2.1. Patients selection

The study was approved by the Clinical Research Ethical Committee of the Chinese University of Hong Kong (approval number CRE-2020.643). All study procedures were in compliance with the Declaration of Helsinki. We retrospectively reviewed all DKD patients who were started on DPP-4 inhibitor in an university teaching hospital from January 1, 2019 to December 31, 2019. All patients were treated by their in-charge physician and the management was not affected by the study. DKD was defined as diabetic patients with urine albumin excretion above 30 mg:g-Cr or urine protein excretion above 0.5 g:g-Cr. Patients who had blood transfusion, recombinant human erythropoietin, iron deficiency, active malignancy, liver cirrhosis, and those receiving sodium-glucose cotransporter-2 (SGLT2) inhibitors or thiazolidinedione therapy during that period were excluded. In addition to the baseline demographic data and the details of DPP-4 inhibitor treatment, we retrieved their complete blood count, serum creatinine, and HbA1c levels from the Hong Kong Hospital Authority Clinical Data Analysis and Reporting System, from 12 months before the initiation of DPP-4 inhibitor treatment to 6 months after the treatment. Most patients had blood test every 2 to 3 months, but the frequency depended on the clinical need. The estimated glomerular filtration rate (eGFR) was calculated by the CKD-EPI equation,^[15]^ and the severity of CKD was classified according to the Kidney Disease: Improving Global Outcomes stages.^[16]^ We also retrieved the baseline clinical and biochemical data of the patients from their electronic patient record.

### 2.2. Outcome measures

The primary outcome was the change in average hemoglobin level from the pretreatment to post-treatment period. The pretreatment period was defined as within 6 months before DPP-4 inhibitor was started, and post-treatment periods as the first 6 months after DPP-4 inhibitor was started (Supplementary Figure 1, http://links.lww.com/MD/J405). As a self-control group, we defined the baseline period as between 6 and 12 months before the DPP-4 inhibitor was started (Supplementary Figure 1, http://links.lww.com/MD/J405).

### 2.3. Statistical analysis

Statistical analysis was performed by IBM SPSS Statistics version 24.0 (IBM corporation, Armonk, NY). The sample size of this study was not calculated but we reviewed all eligible patients in our electronic clinical record database in order to ensure unbiased case selection. Data were expressed as means ± SD. Normality of data was checked by the Shapiro–Wilk Test. Data were compared by paired and un-paired Student *t* test as appropriate. Correlations between continuous variables are calculated by Pearson correlation coefficient. Since the distribution of eGFR was skewed, the Wilcoxon rank sum test and Spearman rank correlation were used for its analysis. Subgroup analysis in patients with available CKD stages and albuminuria stages was performed to investigate the association between the severity of kidney function damage and the change in hemoglobin. A value of *P* < .05 was considered statistically significant. All probabilities were 2-tailed. In order to explore independent factors that affected the change in hemoglobin, we constructed a multiple linear regression model by including factors with *P* value .1 or below in the univariate analysis.

## 3. Results

We reviewed 4133 patients who were started on DDP-4 inhibitor treatment; 443 (10.7%) had complete data on hemoglobin level before and after DPP-4 inhibitor treatment for analysis. The patient flow and reasons of exclusion are summarized in Supplementary Figure 2, http://links.lww.com/MD/J406. The baseline clinical and demographic characteristics for patients with complete hemoglobin profile were compared to that of the entire cohort in Table 1. In short, patients with complete hemoglobin profile was a subgroup with worse kidney function but lower HbA1c within the entire cohort.

**Table 1:** Baseline demographic and clinical characteristics of the entire cohort and patients with complete hemoglobin profile.

	Entire cohort	Complete hemoglobin profile
Total no. of cases	4133	443
Age (yr)	65.5 ± 13.2	67.8 ± 14.1
Gender (M:F)	2045:1843	227:216
HbA1c (%)	8.24 ± 1.46	7.63 ± 1.42
eGFR (mL/min/1.73 m^2^)	75.8 ± 33.8	62.1 ± 36.6
CKD stage, no. of case (%)		
Stage 1	969 (23.4%)	89 (20.1%)
Stage 2	1097 (26.5%)	116 (26.2%)
Stage 3a	415 (10.0%)	72 (16.3%)
Stage 3b	344 (8.2%)	68 (15.3%)
Stage 4	188 (4.5%)	58 (13.1%)
Stage 5	60 (1.5%)	40 (9.0%)
Missing data	1060 (25.6%)	0
Albuminuria, g:g-Cr*	1.80 (0.80–5.20)	2.10 (0.80–5.35)
Albuminuria stage, no. of case (%)		
Stage 1	0	0
Stage 2	429 (10.4%)	87 (19.6%)
Stage 3	2976 (72.0%)	356 (80.4%)
Missing data	728 (17.6%)	0

CKD = chronic kidney disease, eGFR = estimated glomerular filtration rate.

* median (inter-quartile range).

For the 443 patients who had complete hemoglobin profile, there were 216 (48.7%) men, and the mean age was 67.8 ± 14.1 years. Among the 443 patients, 52.4% were treated with vildagliptin, 29.3% with alogliptin, 15.3% with sitagliptin, and 3.0% with linagliptin. Baseline clinical and demographic characteristics of the patients are summarized in Table 2. None of the patient in our cohort received SGLT2 inhibitor or thiazolidinedione therapy.

**Table 2:** Other baseline demographic and clinical characteristics for patients with complete hemoglobin profile.

Total no. of cases	443
Duration of diabetes (yr)	10.9 ± 4.6
Body mass index (kg/m^2^)	22.06 ± 3.96
Blood pressure (mm Hg)	
Systolic	146.0 ± 14.3
Diastolic	81.2 ± 8.4
Peripheral blood count (×10^9^/L)	
White blood cell (n = 384)	7.19 ± 1.96 (NR 4.2–9.6)
Platelet (n = 372)	221.9 ± 64.2 (NR 163–356)
Iron profile	
Plasma iron (µmol/L) (n = 228)	10.15 ± 5.23 (NR 6–35)
Plama TIBC (µmol/L) (n = 226)	43.76 ± 13.29 (NR 41–77)
Iron saturation (%) (n = 226)	24.9 ± 14.5 (NR 15–50)
Serum ferritin (ng/mL) (n = 191)	707.8 ± 956.5 (NR 29–337)
Other nutritional parameters	
Serum vitamin B12 (pmol/L) (n = 252)	425.5 ± 182.0 (NR 179–660)
Serum folate (nmol/L) (n = 252)	30.5 ± 23.2 (NR 7.0–46.4)
LDL cholesterol (mmol/L)	2.95 ± 0.85 (NR < 3.4)
Concomitant medications, no. of case (%)	
Diuretics	221 (49.8%)
RAAS blocker	365 (82.5%)
Statin	163 (36.7%)
Aspirin	186 (41.9%)
Complications and comorbidities, no. of case (%)	
Peripheral neuropathy	76 (17.1%)
Retinopathy	173 (39.0%)
Ischemic heart disease	106 (23.9%)
Congestive heart failure	76 (17.1%)
Cerebrovascular accident	64 (14.4%)
Peripheral vascular disease	28 (6.3%)
Use of anti-diabetic agents, no. of case (%)	
Sulfonylurea	84 (18.9%)
Metformin	175 (39.5%)
Insulin	309 (69.8%)
Iron supplementation, no. of case (%)	38 (8.5%)

CKD = chronic kidney disease, eGFR = estimated glomerular filtration rate, LDL = low density lipoprotein, NR = normal range, RAAS = renin-angiotensin-aldosterone system, TIBC = total iron binding capacity.

### 3.1. Change in hemoglobin after DPP-4 inhibitor

**Figure 1.**
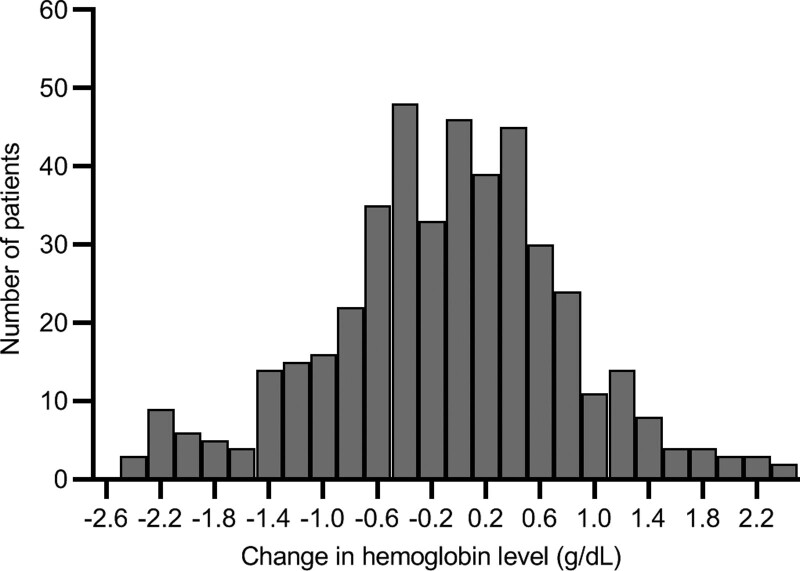
The distribution of the change of hemoglobin level from pretreatment to post-treatment period.

There was a small but significant decline in hemoglobin level from the pretreatment 11.98 ± 2.07 g/dL to post-treatment period 11.87 ± 2.12 g/dL (paired Student *t* test, *P* < .0001). The decline of hemoglobin level in this period was 0.10 ± 0.89 g/dL; with 161 patients (37.2%) had hemoglobin decline ≥ 0.5 g/dL, and 92 patients (21.2%) had a decline ≥ 1.0 g/dL. The distribution histogram of the change in hemoglobin level is shown in Figure 1. There was no significant concomitant change in WBC or platelet count during the study period.

Before the initiation of DPP-4 inhibitor therapy, the average hemoglobin level in the baseline period was 12.22 ± 2.01 g/dL, which showed a significant decline to the pretreatment period (paired Student *t* test, *P* < .0001). The hemoglobin level decreased by 0.24 ± 0.90 g/dL from baseline to pretreatment period, which was significantly more than that from pretreatment to post-treatment period (paired Student *t* test, *P* = .0008). The change in hemoglobin level was not affected by the type of DPP-4 inhibitor (details not shown). We further performed subgroup analysis on the change of hemoglobin level according to the patients’ CKD stage, albuminuria stage, and the use of diuretic therapy, as summarized in Table 3. In essence, the effect of DPP-4 inhibitor on hemoglobin was particularly obvious for patients with CKD stage 5, and those with albuminuria stage 3. In contrast, there was no significant association between patients’ sex, age, presence of comorbid conditions, the use of renin-angiotensin-aldosterone axis inhibitors or other medications, on the change in hemoglobin level (details not shown).

**Table 3:** Subgroup analysis of the change of hemoglobin level.

	n	Hemoglobin level (g/dL)			*P* value		
		Baseline*	Pretreatment*	Post-treatment*	Change #1 (baseline vs pretreatment)	Change #2 (pretreatment vs post-treatment)	Change #1 vs change #2
CKD stage							
1	89	13.20 ± 1.75	13.05 ± 1.85	13.08 ± 1.93	*P* = .090	*P* = .746	*P* = .196
2	116	13.12 ± 1.50	12.87 ± 1.70	12.73 ± 1.71	*P* = .005	*P* = .108	*P* = .407
3a	72	12.46 ± 1.98	12.87 ± 1.70	12.04 ± 1.85	*P* = .046	*P* = .037	*P* = .885
3b	68	11.56 ± 1.72	11.21 ± 1.82	11.29 ± 1.89	*P* = .002	*P* = .441	*P* = .005
4	58	11.15 ± 1.76	10.94 ± 1.73	10.52 ± 1.77	*P* = .072	*P* = .000	*P* = .241
5	40	9.64 ± 1.11	9.32 ± 1.15	9.35 ± 1.32	*P* = .056	*P* = .818	*P* = .179
Albuminuria stage							
2	87	12.21 ± 2.05	11.94 ± 2.15	11.76 ± 2.18	*P* = .005	*P* = .056	*P* = .566
3	356	12.22 ± 2.00	11.98 ± 2.06	11.90 ± 2.11	*P* = .000	*P* = .082	*P* = .035
Diuretic							
no	222	12.25 ± 1.89	11.98 ± 1.98	11.91 ± 2.02	*P* = .000	*P* = .167	*P* = .023
yes	221	12.18 ± 2.12	11.97 ± 2.17	11.84 ± 2.22	*P* = .001	*P* = .048	*P* = .394
Iron supplementation							
no	372	12.22 ± 2.06	11.99 ± 2.09	11.90 ± 2.13	*P* = .000	*P* = .050	*P* = .067
yes	38	12.23 ± 1.71	11.84 ± 1.88	11.53 ± 2.20	*P* = .012	*P* = .032	*P* = .634

CKD = chronic kidney disease. Data are presented as mean ± standard deviation and compared by paired Student *t* test.

* see text for the definitions

### 3.2. Relation with kidney function

The eGFR did not change significantly from pretreatment to post-treatment period (Wilcoxon rank sum test, *P* = .168). The pretreatment eGFR had a substantial correlation with the pretreatment hemoglobin level (*R* = 0.501, *P* < .0001), but did not with the change in eGFR after DDP-4 inhibitor treatment (r = −0.078, *P* = .111).

Before the initiation of DPP-4 inhibitor therapy, the average eGFR in the baseline period was 60.7 ± 36.2 mL/min/1.73m^2^, which showed a significant decline to the pretreatment period (Wilcoxon rank sum test, *P* < .0001). The change in eGFR from pretreatment to post-treatment period was significantly better than that from the baseline to pretreatment period (0.33 ± 14.22 vs −2.29 ± 13.81 mL/min/1.73 m^2^, Wilcoxon rank sum test, *P* = .005). The eGFR at the baseline period correlated with the hemoglobin level at the same time (*R* = 0.538, *P* < .0001), but the change in eGFR from the baseline to pretreatment period did not correlate with the concomitant change in hemoglobin level (*R* = 0.051, *P* = .308). In this study population, there was no significant seasonal effect on the eGFR, hemoglobin level, or their changes in 6 months after DPP-4 inhibitor therapy (Supplementary Table 1, http://links.lww.com/MD/J407). The use of concomitant medications (renin-angiotensin-aldosterone system blocker, diuretics, statin, or aspirin) was not associated with the change in hemoglobin level.

### 3.3. Relation with glycemic control

The HbA1c level decreased significantly from pretreatment to post-treatment period (8.20 ± 1.67% to 7.41 ± 1.39%, *P* < .0001). The change in HbA1c level from pre- to post-treatment period had a modest correlation with the change in hemoglobin level (*R* = 0.218, *P* < .0001). There was no relation between the use of sulfonylurea, metformin, or insulin with the change in hemoglobin level. The relation between change in hemoglobin level and clinical parameters are further explored by multiple linear regression and summarized in Table 4. In this model, only the change in HbA1c from pre- to post-treatment period had independent correlation with the change in hemoglobin level.

**Table 4:** The relation between changes in hemoglobin and clinical parameters.

	Univariate correlation*	Multiple linear regression		
		Unstandardized B	95%CI	*P* value
Baseline eGFR	*R* = 0.070, *P* = .015	0.001	−0.001 to 0.004	*P* = .266
Change in eGFR	r = −0.075, *P* = .124			
Baseline HbA1c	r = −0.132, *P* = .007	-0.022	−0.093 to 0.049	*P* = .546
Change in HbA1c	*R* = 0.218, *P* < .0001	0.098	0.028 to 0.168	*P* = .006
Baseline albuminuria	r = −0.046, *P* = .373			
Change in albuminuria	*R* = 0.060, *P* = .281			

CI = confidence interval, eGFR = estimated glomerular filtration rate.

* Pearson correlation coefficient.

## 4. Discussion

In our study, we found that hemoglobin level gradually decrease with the progression of DKD, and the initiation of DPP-4 inhibitor therapy reduces the rate of hemoglobin decline. The effect of DPP-4 inhibitor on hemoglobin is statistically significant but clinically modest, and did not correlate with the concomitant change in kidney function.

Anemia is a common complication of CKD, and it is particularly common and severe in DKD as compared to non-diabetic CKD.^[8,17,18]^ Anemia may be noticed at stage 3 CKD, and is present in up to 67% to 90% at stage 5.^[19,20]^ With the progressive loss of kidney function, hemoglobin level decreases with time. In the present study, we also found that the hemoglobin level gradually declined from baseline to pretreatment period, but the degree of hemoglobin drop was significantly alleviated following the initiation of DPP-4 inhibitor therapy (i.e., from pretreatment to post-treatment period). From the subgroup analysis, we found that the effect of DPP-4 inhibitor on hemoglobin is particularly prominent in patients with moderate to severe CKD or heavy albuminuria. More importantly, we observed a modest but significant *positive* correlation between the change in HbA1c and hemoglobin levels, indicating that the hematopoietic effect of DPP-4 inhibitor therapy is independent of the improvement in glycemic control. Similarly, the change in hemoglobin level did not correlate with the change in eGFR, suggesting that the hematopoietic effect of DPP-4 inhibitor therapy was not an indirect result of any effect on kidney function.

The physiological mechanism of our observation is not entirely clear, but it is consistent with the findings of basic science studies that demonstrated a link between DPP-4 and hematopoiesis. It is well established that DPP-4 cleaves the N-terminals of granulocyte-macrophage colony-stimulating factor (GM-CSF), interleukin-3, and erythropoietin, and decreases their activity.^[2]^ DPP-4 gene knockout or DPP-4 inhibition improves the activity of these colony-stimulating factors in vitro and in vivo. As compared to the full-length GM-CSF, the DPP-4-truncated GM-CSF has reduced activity due to reduced receptor-binding affinity, ability of inducing GM-CSF receptor oligomerization and its downstream signaling capacity.^[14]^ In the present, we found that DPP-4 inhibitor had no appreciable effect on peripheral white cell or platelet count, suggesting that the effect in human is more prominent on erythropoietin than other colony-stimulating factors. This notion needs to be tested in further studies.

DPP-4 inhibitors have quite heterogeneous chemical structures,^[21]^ and may affect different inhibitions to many enzymes that may affect hemoglobin levels. However, our results showed that the change in hemoglobin level was not affected by the type of DPP-4 inhibitor. A previous clinical trial demonstrated that patients treated with saxagliptin have a unexpected higher incidence of hospitalization for heart failure, this increased risk of heart failure hospitalization was not associated with an increased risk of cardiovascular death or all-cause mortality, the potential explanation is associate with hemodilution.^[22]^ On the other hand, a recent meta-analysis showed that treatment with DPP-4 inhibitors did not significantly increase cardiovascular outcomes in these patients with type 2 diabetes.^[23]^

It is important to note that our study hypothesis was that DPP-4 inhibitor prevents the decline in hemoglobin level in DKD patients. Although hemoglobin level decreased by only 0.1 g/dL within 6 months following DPP-4 inhibitor treatment, the rate of hemoglobin decline in the same group of patients was 0.24 g/dL during the 6 months period before DPP-4 inhibitor therapy was started, indicating that the rate of hemoglobin decline was reduced by nearly 60%. Since the change in hemoglobin level after DPP-4 inhibitor was compared to the change in same group of patients before DPP-4 inhibitor as historical control, the effect of DPP-4 inhibitor appears to be genuine and other patient related factors (e.g., diet and fluid intake) seem unlikely. Nonetheless, we would emphasize although the effect of DPP-4 inhibitor on hemoglobin level is statistically significant, the actual effect is small, and the clinical significance is unknown. The effect of DPP-4 inhibitor on hemoglobin appeared to be particularly marked in CKD stage 3b and 5, but not stage 4, of which the reason is not entirely clear, and further study is deserved. Nonetheless, our study was a proof-of-the-concept and our result confirms an effect predicted by previous laboratory studies,^[14]^ and suggests a new pathway that may be pursued for the treatment of renal anemia. More recently, it is also reported that sodium-glucose transport protein 2 (SGLT2) inhibitors interacts with hypoxia-inducible factors and may promote erythrocytosis.^[24]^ Further studies would also be needed to delineate any additive or synergistic erythropoietic effects between DPP-4 inhibitor and SGLT2 inhibitor, both of which are increasingly used for the treatment of diabetes.^[25]^

In this study, eGFR in the baseline period correlated with hemoglobin levels, but the change in eGFR from the baseline to the pretreatment period did not correlate with the change in hemoglobin level. This observation is consistent with previous reports, which showed that the degree of kidney failure correlates with the severity of anemia, but the rate of kidney function deterioration has only modest correlation with the decline in hemoglobin level.^[8,26]^ Since eGFR had a modest seasonal change in healthy subjects,^[27]^ the observational period should ideally be 12 months, but an extended observational period carries a risk of concurrent problems that may confound the hemoglobin and eGFR levels. Post hoc analysis of our study population also did not show any difference in kidney function, hemoglobin, or their changes following DPP-4 inhibitor between the rainy season (from April to September in Hong Kong) and the dry one (see Table 4).

In addition to the small effect size of DPP-4 inhibitor on hemoglobin level, there are several other inadequacies of our present study. First, we did not perform power calculation for this study, partly because there are few available data to allow sample size estimation. Based on our present result, it could be estimated that around 3200 patients will be required to ensure an adequate sample size to discern a meaningful difference in hemoglobin level for various stages of CKD.^[28]^ Theoretically, the analysis of serum C-reactive protein levels and the change in reticulocyte count before and after DPP-4 inhibitor treatment will further support our hypothesis. Unfortunately, C-reactive protein and reticulocyte count were not a parameter for routine monitoring and the result was available in <10% of our patients. As retrospective study, there was a substantial proportion of missing data in our cohort. Only around 10% patients treated with DPP-4 inhibitors had complete hemoglobin profile for analysis (see Supplementary Figure 2, http://links.lww.com/MD/J406), and this group of patients had better glycemic control and worse kidney function than those with incomplete data (see Table 1), raising the possibility of selection bias in our patient population. On the other hand, neither glycemic control nor baseline kidney function appeared to have any effect on the change in hemoglobin level following DPP-4 inhibitor therapy, suggesting that our observation was a genuine one. It should be noted that the most common reason of exclusion was incomplete data before DPP-4 inhibitor treatment because our center is a tertiary referral one and many patients were started on DPP-4 inhibitor shortly after they were seen in our clinic. Because of the short duration of observation, we do not know whether the effect of DPP-4 inhibitor on hemoglobin level would be short-lasting or progressive. Since the effect of DPP4 inhibitor on hemoglobin was greater in patients with more advanced DKD, follow up studies that focus on stages 3b to 5, as well as on any potential interaction with the use of SGLT2 inhibitor, would be particularly valuable. Furthermore, we did not examine an potential interaction between DPP-4 inhibitor and erythropoiesis-stimulating agent therapy. Given that DPP-4 probably affect hematopoiesis via the cleavage of endogenous erythropoietin, it would be particularly interesting to explore the impact of DPP-4 inhibitor on the efficacy of the new hypoxia-inducible factor prolyl hydroxylase inhibitor, which primarily acts by the stimulation of endogenous erythropoietin production. To prove our hypothesis conclusively, the intact erythropoietin and N-terminal truncated erythropoietin levels before and after DPP-4 inhibitor therapy should be measured so as to prove that erythropoietin was cleaved by DPP-4.^[14]^ Unfortunately the test was not possible because of the retrospective design of our study and lack of archive serum samples. Future studies are needed to test this hypothesis.

## Acknowledgments

The results of this paper was presented as poster in the World Congress of Nephrology, February 2022 (POS 356) and published as an abstract (Zeng LF, Chan GCK, Ng JCK, Szeto CC. DPP-4 inhibitor reduces the worsening of anemia in diabetic kidney disease. Kidney International Reports 2022; 7, S160-162; link: https://doi.org/10.1016/j.ekir.2022.01.377).

## Author contributions

**Conceptualization:** Lingfeng Zeng, Cheuk Chun Szeto.

**Data curation:** Gordon C.K. Chan, Jack K.C. Ng, Winston W.S. Fung.

**Formal analysis:** Lingfeng Zeng, Jack K.C. Ng, Cheuk Chun Szeto.

**Funding acquisition:** Kai-Ming Chow.

**Investigation:** Lingfeng Zeng, Gordon C.K. Chan, Winston WS Fung.

**Methodology:** Lingfeng Zeng, Gordon C.K Chan, Jack K.C. Ng, Winston W.S. Fung.

**Project administration:** Jack K.C. Ng, Kai-Ming Chow.

**Supervision:** Kai-Ming Chow, Cheuk Chun Szeto.

**Writing – original draft:** Lingfeng Zeng.

**Writing – review & editing:** Cheuk Chun Szeto.

## Supplementary Material






