# Supplementary material for: The effect of Dipeptidyl peptidase 4 (DPP-4) inhibitors on hemoglobin level in diabetic kidney disease: A retrospective cohort study
Source: Medicine (Baltimore). 2023 Aug 11;102(32):e34538. doi: 10.1097/MD.0000000000034538 (PMC10419505; doi:10.1097/MD.0000000000034538)

Supplementary Figure 1. Definition of baseline, pre-treatment, and post-treatment periods of the study.

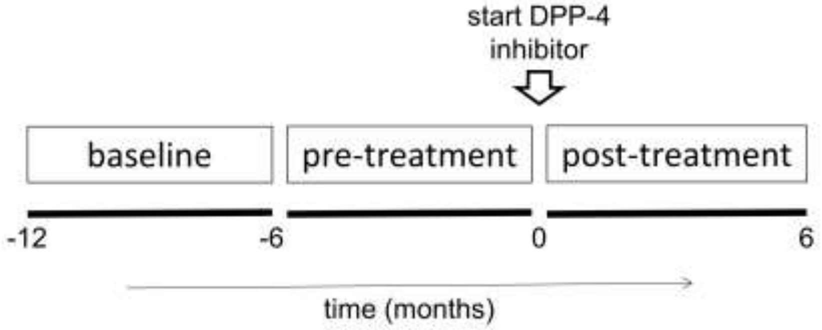

Supplement: Supplementary file 1 [file medi-102-e34538-s001.pdf]
