# Supplementary material for: The effect of Dipeptidyl peptidase 4 (DPP-4) inhibitors on hemoglobin level in diabetic kidney disease: A retrospective cohort study
Source: Medicine (Baltimore). 2023 Aug 11;102(32):e34538. doi: 10.1097/MD.0000000000034538 (PMC10419505; doi:10.1097/MD.0000000000034538)

Supplementary Figure 2. Patient flow and reasons for excluding patients from analysis. (AKI, acute kidney injury)

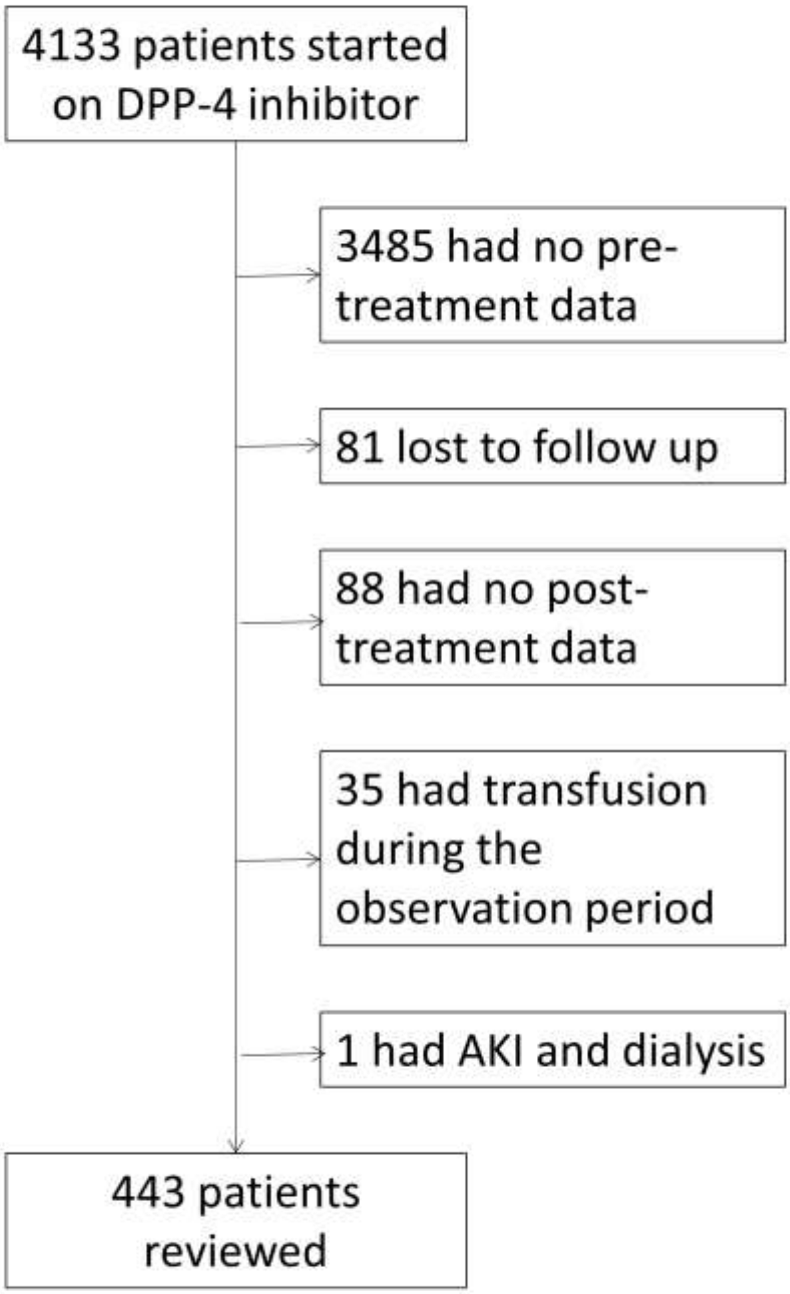

Supplement: Supplementary file 2 [file medi-102-e34538-s002.pdf]
