# Supplementary material for: The effect of Dipeptidyl peptidase 4 (DPP-4) inhibitors on hemoglobin level in diabetic kidney disease: A retrospective cohort study
Source: Medicine (Baltimore). 2023 Aug 11;102(32):e34538. doi: 10.1097/MD.0000000000034538 (PMC10419505; doi:10.1097/MD.0000000000034538)
Supplement: Supplementary file 3 [file medi-102-e34538-s003.pdf]

Supplementary Table 1. Effect of climatic change on kidney function and hemoglobin level

	Rainy season	Dry season	P value
no. of patients	255	188	
eGFR (ml/min/1.73m^2)	57.7 ± 34.7	62.0 ± 37.4	p = 0.224
GFR change in 6 months	-0.3 ± 12.2	1.0 ± 16.7	p = 0.358
hemoglobin (g/dL)	11.9 ± 2.1	12.1 ± 2.0	p = 0.352
hemoglobin change in 6 months	-0.11 ± 0.86	-0.08 ± 0.93	p = 0.714
